# Diabesity and Dietary Interventions: Evaluating the Impact of Mediterranean Diet and Other Types of Diets on Obesity and Type 2 Diabetes Management

**DOI:** 10.3390/nu16010034

**Published:** 2023-12-21

**Authors:** Eleni Pavlidou, Sousana K. Papadopoulou, Aristeidis Fasoulas, Vasileios Papaliagkas, Olga Alexatou, Maria Chatzidimitriou, Maria Mentzelou, Constantinos Giaginis

**Affiliations:** 1 Department of Food Science and Nutrition, School of the Environment, University of the Aegean, 81400 Lemnos, Greece; elen.p.pavl@gmail.com (E.P.); athanarist@gmail.com (A.F.); rd.olga.alexatou@gmail.com (O.A.); maria.mentzelou@hotmail.com (M.M.); cgiaginis@aegean.gr (C.G.); 2 Department of Nutritional Sciences and Dietetics, School of Health Sciences, International Hellenic University, 57400 Thessaloniki, Greece; 3 Department of Biomedical Sciences, School of Health Sciences, International Hellenic University, 57400 Thessaloniki, Greece; vpapaliagkas@gmail.com (V.P.); mchatzid952@gmail.com (M.C.)

**Keywords:** diabesity, obesity, diabetes mellitus, glycemic control, weight loss, dietary interventions, Mediterranean diet, low-carbohydrate diets, nutritional strategies, metabolic health

## Abstract

Background: Diabesity, the intersection of obesity and diabetes, presents a global health crisis with profound implications. Addressing diabesity requires multifaceted strategies, with diet playing a pivotal role. Over the last 15 years, clinical studies have intensified their exploration of various dietary approaches in diabesity management. This literature review aims to synthesize findings from clinical studies conducted in the last 15 years, shedding light on the efficacy, mechanisms, and nuances of different diet types in diabesity management with special focus on the Mediterranean diet (MD). Methods: Thorough research of academic databases yielded a collection of relevant clinical studies. These studies encompassed a range of dietary strategies, including the MD, low-carbohydrate diets, plant-based diets, high-protein diets, low-fat regimens, and intermittent fasting. Key findings, methodologies, and outcomes were thoroughly extracted and analyzed. Results: The last 15 years have witnessed considerable improvements in recognizing the role of human nutritional habits in diabesity management. The MD appears to be the most well-recognized diet, exerting favorable effects against both obesity and diabetes. Low-carbohydrate diets were found to enhance glycemic regulation and decrease insulin resistance. Plant-based diets demonstrated potential benefits in weight management and cardiometabolic health. High-protein, low-fat dietary models exhibited positive effects on satiety and body weight decline. Intermittent fasting regimens also exerted metabolic improvements and body weight decline. Personalization emerged as a crucial factor in dietary recommendations. Conclusions: Clinical studies from the last 15 years underscore the intricate relationship between diet types and diabesity management. The above results contribute to an increasing body of evidence, emphasizing the need for tailored dietary approaches and especially the MD. Healthcare providers can utilize this knowledge to offer personalized dietary recommendations for individuals with diabesity, potentially curbing the rise of these twin epidemics and improving the well-being of affected populations.

## 1. Introduction

In the modern era, the convergence of two formidable health challenges has spurred a paradigm shift in healthcare regarding diabesity. Coined to reflect the intricate interplay between diabetes and obesity, diabesity has emerged as a global health crisis of unprecedented proportions [1,2]. With soaring prevalence rates, diabesity exacts a profound toll on individuals’ well-being, healthcare systems, and economies worldwide [2]. Addressing this complex syndrome requires innovative and multifaceted approaches, with dietary interventions at the forefront [2].

**Figure 1:**
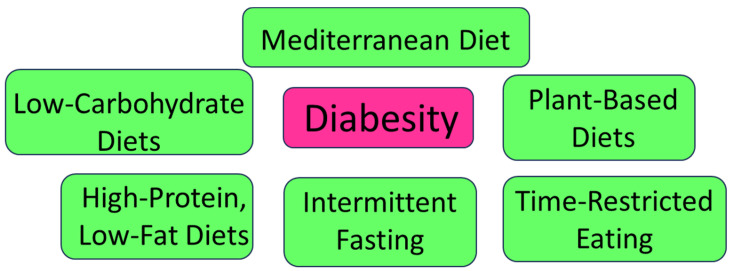
Diabesity and diverse dietary interventions.

Over the last 15 years, there has been a surge in clinical studies examining the impact of various diet types on diabesity management. These studies have provided a rich tapestry of evidence, offering insights into the nuanced relationship between dietary choices and diabesity progression [3,4]. From low-carbohydrate regimens to plant-based diets, intermittent fasting, and high-protein, low-fat approaches, the dietary landscape for diabesity management has never been more diverse (Figure 1) [3,4,5]. Notably, adherence to the Mediterranean diet (MD) has been considered as the most recognized and well-studied diet, which is highly related to a lowered probability of various chronic diseases, including metabolism-related diseases like diabetes mellitus as well as obesity [6,7]. The MD is not restricted to exacting healthful nutritional habits. It further contains specified abilities, knowledge, practices, symbols, and traditions concerning crops, harvesting, fishing, animal farming, conservation, managing, cooking, and, particularly, the allocation and intake of foods patterns [8,9].

This comprehensive literature review embarks on a journey through the expansive realm of diabesity and diet types. Our aim is to synthesize the latest findings from clinical studies conducted in the past decade, shedding light on the efficacy, mechanisms, and nuances of different dietary strategies in the context of diabesity [4,10]. Through this exploration, we seek to equip healthcare professionals, researchers, and individuals grappling with diabesity with a holistic identifying of the impact that nutrition can exert concerning its management.

The global prevalence of diabesity paints a stark picture. Based on the International Diabetes Federation (IDF), more than half of all adults worldwide, about one billion, were diagnosed with diabetes in 2021, with projections indicating a staggering rise to 700 million by 2045 [1]. Simultaneously, the World Obesity Federation highlights that obesity affects nearly 2.8 billion individuals worldwide [2]. However, it is at the intersection of these two epidemics that the most significant health challenges arise. The coexistence of obesity and diabetes significantly elevates the probability of morbidity, such as cardiometabolic disorders, stroke, kidney disorder, and certain cancers [11]. Furthermore, diabesity often manifests as a syndrome of insulin resistance, where the body’s cells are more resistant to insulin, resulting in elevated blood glucose amounts [12]. As a result, effective management necessitates comprehensive strategies that encompass both weight control and glycemic regulation [13].

The link between diet and diabesity management is not a recent revelation. For decades, healthcare professionals have recognized the pivotal impact of diet in shaping the trajectory of the above states [14]. However, the last ten years have witnessed an explosion of research endeavors aimed at unraveling the complexities of dietary interventions [15]. This period has been marked by advancements in study design, the incorporation of cutting-edge technologies, and an ever-deeper understanding of the molecular underpinnings of diabesity [16]. This review embarks on a systematic exploration of clinical studies published over the last fifteen years [12,17]. These studies encompass a wide spectrum of dietary approaches, each offering unique insights into diabesity management. From elucidating the effect of low-carbohydrate dietary models on glycose regulation for unraveling the success of intermittent fasting regimens, we delve into the scientific literature to distill key findings [11,12].

The dietary landscape we traverse includes the MD, low-carbohydrate diets, plant-based diets, high-protein, low-fat regimens, and intermittent fasting [18,19]. Each of these dietary strategies presents a distinctive profile of macronutrient composition, metabolic effects, and potential benefits in diabesity management [18,19]. Moreover, we examine the importance of individualization in dietary recommendations, recognizing that there are no general rules and approaches to diabesity [20,21].

In navigating the complex terrain of diabesity and diet types, our review aims to empower healthcare providers with evidence-based insights to tailor dietary recommendations to individual needs [22,23]. Equally important is the potential impact of dietary choices on preventing diabesity in high-risk populations, thereby curbing the relentless rise of these twin epidemics [22,23]. As we embark on this journey through the scientific literature, we invite readers to join us in unraveling the intricate connections between diabesity and diet. Together, we will explore the latest research findings, illuminate the pathways to improved diabesity management, and ultimately strive to alleviate the burden of this global health crisis.

## 2. Methods

To conduct this thorough literature review, a comprehensive search was performed across multiple academic databases, such as Medline, Scopus, Web of Science, and Google Scholar. The research was directed to clinical surveys performed in the previous 15 years, from 2008 to 2023. The search terms included various combinations of keywords related to “diabesity”, “obesity”, “diabetes”, and “diet”. Boolean operators like “AND” and “OR” were employed to refine the research and ensure inclusivity. The inclusion criteria for selecting relevant surveys were: (1) clinical surveys presented in peer-reviewed journals, (2) surveys performed on humans, and (3) surveys published between 2008 and 2023. Clinical human studies examining the effects of different diet types on diabesity, including the MD, low-carbohydrate diets, plant-based diets, high-protein, low-fat diets, and intermittent fasting were included. Studies reporting relevant outcomes related to glycemic control, insulin resistance, weight management, and metabolic parameters were also included. Exclusion criteria encompassed: (1) non-clinical studies, such as animal experiments or in vitro research, (2) studies published before 2003, (3) studies not related to diet or diabesity, and (4) studies with inadequate reporting of methodologies or outcomes.

Upon identifying relevant studies, data extraction was carried out systematically. Key information was extracted, including study design, sample size, participant characteristics, duration of intervention, dietary interventions, primary outcomes, and key findings. The data were then analyzed to identify common themes, trends, and significant findings across the selected studies. Particular attention was paid to variations in dietary approaches and their impact on diabesity-related parameters. The quality of the selected surveys was evaluated utilizing appropriate methods, like the Cochrane Risk of Bias Tool for randomized controlled trials (RCTs) and the Newcastle-Ottawa Scale for observational surveys, which were categorized as having low, moderate, or high probability of biases based on their methodology, reporting, and potential sources of bias. The results obtained by the selected surveys were synthesized to create a thorough overview of the efficacy, mechanisms, and nuances of different diet types in diabesity management. The synthesis included a discussion of quantitative and qualitative results, allowing for a nuanced understanding of the relationships between diet and diabesity. This literature review relied solely on evidence derived by previously published surveys. Thus, ethics authorization was not needed for this research.

## 3. Results

### 3.1. Mediterranean Diet and Diabesity

The MD includes traditional dietary patterns of countries bordering the Mediterranean Sea, and it has gained significant attention for its potential benefits in managing diabesity—the intricate interplay of obesity and type 2 diabetes [24,25]. The MD includes an increased intake of fruits, vegetables, whole grains, legumes, and nuts, with a modest consumption of fish and poultry. Additionally, it is notable for its use of extra virgin olive oil as the primary source of nutritional fats. However, variations may exist in the types and quantities of fruits and vegetables consumed across the different studies examining the MD [24,25]. Addressing this variability, we carefully reviewed the currently available studies related to diabesity and cited in our manuscript to identify potential differences in the implementation of the MD. While the fundamental principles remain consistent, it is acknowledged that specific studies may have variations in the recommended portions and types of fruits and vegetables [24,25]. Furthermore, we recognize the significance of extra virgin olive oil in the MD and acknowledge its mention in our manuscript. However, to provide a thorough overview, we have scrutinized the cited studies to confirm the utilization of extra virgin olive oil in each investigation. It is imperative to note that not all studies may explicitly specify the kind of olive oil examined; nevertheless, the prevailing emphasis on extra virgin olive oil within the broader context of the MD is acknowledged. By addressing these points, we aim to enhance the clarity and transparency of our discussion on the MD and its impact on diabesity.

A comprehensive literature search emphasized the interdisciplinary collaboration between endocrinologists and nutritionists to obtain the best possible management of endocrinal diseases, such as the possible impact of the MD concerning their preventive and confronted approaches against these [26]. The above evidence suggested that the MD could exert a preventing impact on endocrinal diseases as well as its introduction into nutritional proposals, which could be proved favorable [24]. A review of evidence concerning healthy effects of MD theories and compliance in a planeterranean viewpoint documented that the MD represents a healthful and sustainable lifestyle model, which can exert preventing effects against diverse disorders, decreasing early mortality [25]. Moreover, accessibility to front-of-pack labels, such as MD principles, could encourage further awareness of food selections amongst customers, promoting both human and planetary health [27]. 

MD, celebrated for its emphasis on whole, nutrient-dense foods, has emerged as a dietary pattern of concern in lowering the probability of diabesity. The study by InterAct Consortium (2011) investigated the link of MD compliance with the risk of insulin-independent diabetes [26,27,28]. This extensive European study found a notable association: individuals who adhered closely to the MD experienced a reduced probability of insulin-independent diabetes. The above provides evidence for adopting the MD, which could serve as a preventive measure against diabesity [28,29,30]. 

The Mediterranean diet’s focus on whole grains, fruits, vegetables, and healthy fats positions it as a holistic approach to managing diabesity, intertwining both diabetes and obesity management into a unified dietary strategy. Notably, high MD compliance, and especially seafood consumption, which are rich in omega-3 unsaturated lipid acids, has been related to lowered insulin resistance in overweight and obese persons [31]. The consumption of dietary fibers, unsaturated fatty acids, vitamins, and polyphenols—such as flavonoids and stilbenes—has been associated with the beneficial effects of the MD against central adiposity, hyperglycemia, hyperlipidemia, and hypertension. Antioxidant and anti-inflammation activities of polyphenols in conjunction with the impacts of unsaturated fatty acids on lipid metabolism contribute to the underlying mechanisms. Overall, dietary interventions using MD components appear to improve metabolic syndrome health biomarkers in humans and/or rodents [32]. 

Moreover, a high compliance to a well-balanced, healthy dietary pattern related to the Mediterranean diet lifestyle could control hyperglycemia, providing beneficial effects to body composition, which may contribute to the management and the prevention of the development of T2DM [33]. The up-coming Prevención con Dieta Mediterránea-Plus (PREDIMED-Plus) RCT has supported that a nutritional intervention related to the weight-decline lifestyle based on an energy-decreased MD in conjunction with elevated physical activity substantial decreased overall and central adiposity and ameliorated age-associated declines of lean mass in overweight or obese older adults suffered from metabolic syndrome. However, continued follow-up is recommended to verify the long-term effects of the above alterations on cardiovascular disease symptomatology [34]. A taste-oriented nutritional interventional study with a duration of one month after a controlled balanced MD was considered as an efficient remedy, which considerably improved all the anthropometry and blood biomarkers. Thus, a taste-oriented MD nutritional intervention was identified as a promising strategy to develop more personalized, taste-oriented follow-up interventional studies to retain a balanced and long-standing body weight decline [35]. 

A UK Biobank cohort conducted on 112,493 people without any cardiometabolic disease and insulin-independent diabetes in the age range of 40–69 years clearly indicated that greater MD compliance was related to decreased probability of diabetes within the UK Biobank. Additionally, Mediterranean-type lifestyle, culturally followed by non-Mediterranean peoples, may promote diabetes prevention [36]. In PREDIMED (“PREvención con DIeta MEDiterránea”), a Spanish clinical study conducted on 7447 men and women, assessed 3541 participants at high cardiovascular risk and primarily without diabetes [30]. The participants were initially randomly assigned to one of three diets: a low-fat diet (n = 1147, control group), MD enhanced with extra virgin olive (n = 1154), or MD accompanied by diverse nuts (n = 1240) [30]. This study showed a convincing inverse linear association of MD with insulin-independent diabetes mellitus [30]. Another RCT compared high-versus low-Glycemic Index nutritional interventions, including 156 adults at risk of insulin-independent diabetes for 3 months [37]. This study indicated that women were more vulnerable to the metabolic impacts of the dietary Glycemic Index compared to men [37]. The above study is characterized by a clinically and scientifically robust relevance and, if verified in future clinical trials, it may reveal significant perspectives for nutritional policies for diabetes and cardiovascular disorder prevention in the setting of individualized diets [37]. In addition, the MedLey trial, a parallel design, population-based, 6-month partial-feeding RCT showed that an objectively assessed MD adherence may be related to decreased probability of insulin-independent diabetes and that even somewhat greater MD compliance could enhance the likelihood of decreasing the incidence of insulin-independent diabetes at a population basis significantly [38].

A cross-sectional survey performed on 346 individuals at risk of prediabetes showed that a greater scoring on the 14-item MEDAS screener was considerably related to a decreased likelihood of pre-diabetes [39]. The participants diagnosed with insulin-independent diabetes presenting modest MD compliance showed greater physical activity levels compared to participants presenting low MD adherence. The quality of life was also elevated with normal training in conjunction with the beneficial impacts of MD compliance on human health [40]. A pilot RCT demonstrated that MD-based nutritional intervention in children with pre-diabetes in a rural region showed that the MD significantly decreased glycated hemoglobin in children with prediabetes compared to control group [41]. A cross-sectional study including 65 individuals indicated that greater MD compliance was related to reduced insulin tolerance and certain inflammatory biomarkers, especially in overweight and obese persons who were not diagnosed with diabetes. Higher MD adherence was associated with greater insulin sensitivity after adjustment for age, gender, and body fat content, and reduced NF-κB activity, as well as elevated adiponectin and adipsin levels after adjustment for potential confounders [42]. A meta-analysis including five RCTs showed that the MD considerably decreased the frequency of gestational diabetes mellitus (GDM), Homeostatic Model Assessment for Insulin Resistance (HOMA-IR), insulin treatment, and gestational weight gain (GWG) than the regular intervention for pregnant mothers [43]. 

A longitudinal birth cohort survey in Iran, including 647 pregnant women, showed that greater MD compliance during early gestation could be related to a decreased probability of GDM, highlighting the need of performing population-based surveys to verify the above results [44]. A MD-based intervention study conducted at the first weeks of gestation revealed permanent positive impacts on abnormal glucose regulation and metabolic syndrome rates at 3 years after delivery [45]. A longitudinal study of pregnant mothers enrolled at the initial three months of gestation indicated that a higher MD compliance before gestation, particularly with fewer meat intake, could exert a protective impact on the incidence of GWG [46]. In another longitudinal, multicenter survey conducted on 7798 pregnant women, a high versus a low a MD scoring was related to a 37% reduced risk of GDM [47]. 

In our exploration of the impact of the MD on diabesity, the importance of maintaining a high level of compliance emerges as a crucial factor influencing the attainment of long-term benefits. In the present review, we underscore the significance of sustained adherence to the MD for optimal effects in the controlling of obesity and insulin-independent diabetes [48]. In this aspect, the literature suggests that successful and lasting adoption of the MD is influenced by several key elements, including education, cultural factors, social support, and individual preferences [49]. More to the point, several studies have highlighted the role of educational interventions in promoting awareness and understanding of the MD’s health benefits, thereby fostering a sense of commitment among individuals [49,50]. Cultural factors, such as the integration of traditional dietary practices into daily routines, have also been associated with sustained adherence [50]. Moreover, the availability of social support networks through community programs or familial encouragement plays a pivotal role in reinforcing positive dietary habits. The customization of dietary recommendations based on individual preferences and lifestyles further enhances the likelihood of continued MD adherence [51].

### 3.2. Low-Carbohydrate Diets and Diabesity

Low-carbohydrate dietary models have currently attracted raising interest as a probable dietary strategy for managing diabesity, a challenging combination of diabetes and obesity. The popularity of low-carbohydrate dietary models in the previous few years has provoked numerous research surveys to investigate their effect in diverse metabolic and non-morbidity states [52]. In a groundbreaking clinical survey, researchers investigated the impacts of a low-carbohydrate diet on weight decline and diabetes control in individuals with insulin-independent diabetes [53]. This study revealed compelling results, demonstrating that participants on a low-carb dietary model exhibited not only significant weight loss but also remarkable improvements in their diabetes control parameters, including HbA1c levels and fasting blood glucose levels [53]. These findings highlight the beneficial potential of low-carbohydrate dietary models in addressing diabesity-related concerns.

Supporting this, Westman et al. (2008) performed a survey examining the impact of a low-carbohydrate, ketogenic dietary model on glycemic control in individuals with insulin-independent diabetes mellitus [54]. The ketogenic diet, including a high fat and low carbohydrate content, showed promising results. Participants following this diet exhibited improved glycemic control, further emphasizing the potential of low-carb diets as a means to manage diabetes while simultaneously addressing obesity [54].

Low-carbohydrate diets, often criticized for their sustainability and long-term effects, are increasingly recognized for their role in helping individuals with diabesity achieve weight loss and improved blood sugar control [55]. These insights pave the way for personalized dietary interventions tailored to the unique needs of those with diabesity. In this aspect, individuals with diabesity adopting a low-carbohydrate diet for 6 months could experience a reduction in diabetes symptoms without adverse effects. Limitations provoke continuous debate about what is considered a remission of diabetes, as well as the efficiency, protection, and nutritional enjoyment of prolonged low-carbohydrate diets [55].

### 3.3. Plant-Based Diets and Diabesity

Plant-based dietary models, depicted by their heavy reliance on fruits, vegetables, whole grains, and legumes, exhibit considerable interest for their probable healthy effects. Kahleova et al. (2020) carried out a study exploring the effects of a low-fat vegan diet on various health parameters in overweight adults [56]. Their results showed that participants in this plant-based diet experienced not only weight loss but also improved insulin sensitivity, a crucial factor in diabetes management [56]. The above results emphasize the potential of plant-based diets in managing diabesity by addressing both obesity and diabetes.

Expanding on the concept of plant-based diets, Ghaedi et al. (2019) performed a systematic review and meta-analysis focusing on the Paleolithic diet, which emphasizes whole, unprocessed foods similar with those found in a plant-based diet [57]. The findings of their study showed that the Paleolithic diet may be effective in improving cardiovascular risk factors, including those associated with diabesity [57]. This suggests that plant-based and whole-food diets not only offer potential benefits for diabetes management but also contribute to overall cardiovascular health, a crucial aspect in diabesity prevention. Epidemiological surveys constantly indicate reduced incidence of mortality in adults adopting plant-based diets in comparison with individuals whose diet systematically contains meat. Plant-based diets have been related to several healthy effects such as a better metabolic and inflammatory profile [58].

These studies collectively underscore the potential of plant-based and whole-food diets in positively influencing diabesity-related factors, such as lipid profiles and cardiovascular health, and further highlighting their role as comprehensive dietary strategies for diabesity management. In total, the health of individuals adopting plant-based diets seems to be usually good, with certain strengths but also with some disadvantages, and the extent to which the disadvantages could be alleviated by best possible foods’ choices, fortification and supplementation has not elucidated yet [59]. The attention in probable longevity-promoting mechanisms of plant-based diets has been enlarged in the last few years since several features of plant-based diets like protein limitation as well as the reduction in specific amino acids that are recognized to increase one’s lifespan [58].

In addressing the potential concerns related to plant-based diets, it is essential to dispel common misconceptions and provide a more nuanced understanding. Non-specialists might perceive plant-based diets as having potential issues such as inadequate protein intake and excessive consumption of sugars, particularly fructose. In this aspect, it should be emphasized that well-planned, plant-based diets can meet protein requirements through diverse plant protein sources, containing legumes, tofu, tempeh, nuts, and seeds. Adequate planning ensures that individuals following a plant-based diet receive a balanced and sufficient intake of essential amino acids [60]. Moreover, concerns about excessive sugars, especially fructose, can be mitigated by emphasizing whole, unprocessed plant foods. Fruits, vegetables, whole grains, and legumes are integral components of a plant-based diet and provide essential nutrients without the added sugars commonly found in processed foods. It is crucial to highlight that the emphasis should be on consuming whole, nutrient-dense foods rather than relying on processed plant-based alternatives [61].

### 3.4. High-Protein, Low-Fat Diets and Diabesity 

High-protein, low-fat diets have gained attention for their potential in addressing diabesity. In a survey of Layman et al. (2015), the researchers explored the impact of a high-protein, low-fat diet on weight decline and glycemic control in individuals with obesity and insulin-independent diabetes [62]. The results were noteworthy, with participants experiencing significant weight decline and decrease in insulin tolerance [62]. This suggests that a nutritional approach, including higher levels in protein and lower levels of in fat may serve as a valuable strategy for managing diabesity by addressing both diabetes and obesity simultaneously.

In comparison with a typical-protein, low-fat diet, a high-protein, low-fat diet produced more advantageous alterations in weighted mean differences for decreases in body weight, fat mass, and triglycerides and attenuations of decreases in fat-free mass and resting energy expenditure. Alterations in fasting plasma glucose, fasting insulin, blood pressure, and total, LDL, and HDL cholesterol were analogous with nutritional interventions. Moreover, greater satiety with a high-protein, low-fat diet was reported in most existing studies [63].

Moreover, Guldbrand et al. (2012) performed a RCT to examine the impact of a low-carbohydrate, high-protein diet in individuals presenting insulin-independent diabetes [56]. This study demonstrated that participants on the high-protein diet achieved better glycemic control and reduced medication usage [64]. This underscores the potential of high-protein, low-fat diets in diabetes management and highlights their role in reducing diabesity-related complications. Thus, high-protein, low-fat diets, characterized by their focus on lean protein sources and limited fat intake, offer a promising avenue for individuals seeking comprehensive diabesity management.

Regarding the potential concerns associated with high-protein, low-fat diets, it is essential to address the issue of kidney load and provide clarity on the upper limit of protein intake and its sources. Concern about kidney load is a valid consideration, particularly when implementing a diet rich in protein. While high-protein diets have shown favorable effects on weight loss, glycemic control, and satiety, it is crucial to understand the upper limit of protein intake that can be safely consumed without adverse effects on kidney function [65]. In this aspect, it has been indicated that the source of protein may exert a considerable effect in mitigating the potential impact on the kidneys. If the diet is predominantly high in plant-based proteins, such as those derived from legumes, tofu, and nuts, the renal load may differ from diets high in animal-based proteins. Plant-based proteins tend to be accompanied by beneficial components such as fiber and antioxidants, which may contribute to a more favorable renal profile [66].

### 3.5. Fasting and Diabesity 

Intermittent fasting and time-restricted eating have been considered as intriguing dietary strategies for diabesity management. Intermittent fasting increased in accordance with its popularity on the internet. Intermittent calorie restriction/time-restricted eating is a kind of calories’ reduction turning around a small period of feeding and a relatively higher period of fasting. The above model of feed–fast cycle stimulates an elevated reduction in adipose tissue and glycogen stores, resulting in higher fatty mass decline and decreased saturation. Intermittent fasting may also exert cardioprotective effects, controlling diabetes-related characteristics, as well as reducing the prevalence of diabetes [67].

In another survey by Herpich et al. (2022), researchers investigated the impact of time-restricted eating on insulin tolerance and cardiometabolic health [58]. The findings indicated that participants who practiced time-restricted eating experienced greater insulin sensitivity and reduced insulin amounts, suggesting its potential in managing diabetes, a critical aspect of diabesity [68]. Intermittent fasting appears to affect, in a different way, metabolic homeostasis, which is dependent on a personalized basis on one’s health condition and the kind of metabolic disorder [69]. Various modes of intermittent fasting decrease body weight and lowered diabetes parameters such as fasting glucose and insulin, HOMA-IR index, and glycosylated hemoglobin (HbA1c) [70].

Further supporting the role of fasting, Halberg et al. (2005) performed a survey on the impact of intermittent fasting on body weight and insulin resistance [71]. The results revealed significant body weight decline, decreasing insulin resistance in participants practicing intermittent fasting [61]. These findings underscore the potential of fasting regimens in addressing both obesity and diabetes, key components of diabesity. The concept of fasting, whether through intermittent fasting or time-restricted eating, presents a novel approach to diabesity management that warrants further exploration and customization to individual needs.

Moreover, Christian Orthodox fasting constitutes a dietary model with increased complex carbohydrates’ content and decreased levels of refined carbohydrates. This kind of fasting has been explored in relation to its probable healthy effects. In fact, it is characterized by positive impacts regarding glucose and lipid control; however, its impact against hypertension remains inconclusive [72]. Regarding body weight monitoring, Christian fasters showed decreased body mass and lesser calories intake during fasting days. More to the point, Christian fasters consume increased amounts of fruits and vegetables without nutritional deficiencies for iron and folate [72].

There are also data which indicate that short-term intermittent fasting exerts a higher beneficial impact in animal studies and promotes effects benefits in RCT [73]. However, there are not adequate surveys conducted on individuals affected by obesity and insulin-independent diabetes. It also remains a debatable interventional approach for the treatment of metabolic disorders and cancer. Moreover, whether intermittent fasting could be used to long-term clinical treatment, and whether it exhibits adverse effects during the long-term period or not, requires further large-scale and long-term studies [73].

Incorporating high-protein, low-fat diets and fasting into the discussion expands the repertoire of dietary strategies available for diabesity management, providing individuals with diverse and potentially effective approaches to address this complex condition. High-protein, intermittent fasting, and a low-calorie diet are related to analogous reductions in BMI and blood lipids in individuals affected by obesity. This kind of dietary model also shows an improvement in reducing body weight regain, improving arteries health as compared to a heart healthy diet over a period of one year [74]. Moreover, the findings of the currently available clinical studies have demonstrated that LCDs and intermittent fasting in obese patients (containing those with simultaneous insulin independent diabetes) may result in body fatty mass decline and improvements in metabolic parameters. The positive impacts mentioned above may be ascribed not only to body mass decline, but additionally to the triggering of metabolic pathways related to fasting conditions. Nevertheless, the lack of large-scale RCTs makes it difficult to propose LCDs or intermittent fasting as consistent, habitual practices for effective and permanent weight decline [75].

As far as fasting strategies for diabesity management are concerned, it is pertinent to take into account the nutritional aspects of post-fasting periods. While intermittent fasting and time-restricted eating show promising impacts on insulin sensitivity, cardiometabolic health, and weight management, the question of what should be ingested after the fasting period is a crucial aspect that merits discussion. In this context, post-fasting nutrition plays a pivotal role in optimizing the benefits gained during the fasting phase. The choice of nutrient-dense, well-balanced meals after a fasting window can influence metabolic outcomes and overall health. Several studies have indicated that incorporating a balanced combination of macronutrients, including lean proteins, complex carbohydrates, and healthy fats, can help sustain the positive effects of fasting, support muscle recovery, and regulate blood glucose levels [76].

Moreover, it should emphasize the significance of avoiding excessive consumption of refined sugars and extremely processed foods during post-fasting periods. These dietary choices may counteract the benefits of fasting by contributing to insulin resistance and metabolic dysregulation [77]. In light of these considerations, we acknowledge the significance of addressing post-fasting nutrition. While the existing literature provides insights into the positive effects of fasting, further research is needed to delineate specific dietary recommendations for optimal post-fasting nutrition. This area remains a dynamic and evolving field, and future studies exploring the interplay between fasting strategies and post-fasting dietary choices will contribute valuable information to refine dietary guidelines for individuals practicing of intermittent fasting or time-restricted eating [70,78,79,80,81,82,83,84,85,86,87,88,89]

## 4. Discussion

Comprehensive analysis of clinical trials conducted across the past years has revealed a complex interplay between diet types and diabesity management. The findings of these studies contribute valuable insights to the ongoing discourse surrounding dietary strategies for individuals with diabesity. 

The MD is a plant-based, high-unsaturated fat nutritional model, which has been constantly related to decreased prevalence of non-communicable disorders and overall mortality in longitudinal surveys and with decreased CVD in the PREDIMED trial. For these merits above other diets, this nutritional model may further be applied favorably for weight decline [80]. It has also been well-established that the MD decreases the prevalence and the development of insulin-independent diabetes and its relationship with diverse cardiovascular healthy diets. The MD can exert a crucial impact in insulin-independent diabetes-related mechanisms due to the fact that it includes several anti-inflammatory or antioxidant ingredients, glucagon-like peptide agonist molecules, and alterations in intestinal microbiome. Every ingredient of the MD may be implicated in processes associated with diabetes homeostasis, and some of them share similar physio-pathological paths. The significance of this nutritional model surrounded by specific habits of a healthy lifestyle should be highlighted [81]. Moreover, adopting a Mediterranean-style diet is greatly supported due to its healthy ingredients such as polyphenols. Indicatively, hydroxycinnamic derivatives, quercetin, resveratrol, oleuropein and hydroxytyrosol that are well-recognized for their antioxidant and anti-inflammatory activities, exhibited also anti-obesity functions [82].

Moreover, the MD has successfully been recognized as an effective regulator of the intestinal microbiome related to increased intake of plant-based foods, modest consumption of seafoods and dairy products, and decreased red meat intake. An inverse association of MD compliance has also been established with chronic disorders, including obesity and diabetes. The MD may also be helpful in the prevention of the pathogenesis of the above disorders because of its impacts on the gut microbiome. Indicatively, it has been reported that the amount of Bifidobacterium and Bacteroides increase the longer one’s consumption routine adheres to the MD, while the amount of Firmicutes reduces, reinforcing the symbiotic distribution in the gut microbiome [83].

Numerous clinical trials have investigated the efficacy of low-carbohydrate diets, including ketogenic diets, in diabesity management [84,85,86,87]. These studies consistently report improvements in glycemic control, reduced insulin resistance, and notable weight loss among participants. The low-carbohydrate approach leads to a condition of ketosis, where the body relies on fat as a primary energy source. This metabolic shift may enhance insulin sensitivity and promote fat loss [86]. However, concerns regarding long-term adherence and potential adverse effects, such as nutrient deficiencies and cardiovascular risks, must be addressed [88].

Low-carbohydrate diets have attracted attention because of their potential to improve glycemic control and promote weight decline in individuals with diabesity [52,89]. By restricting carbohydrate intake, these diets can lower postprandial glucose levels and reduce insulin resistance, which are critical factors in managing type 2 diabetes [90]. However, it is important to consider the quality of carbohydrates in the diet, as whole grains and fiber-rich foods should not be indiscriminately eliminated [91].

Plant-based diets have also attracted attention for their potential to mitigate diabesity-related risks [92,93,94]. These dietary patterns include high amounts of fruits, vegetables, whole grains, and legumes, offering diverse vitamins, minerals, and antioxidants. Clinical studies have consistently showed improvements in insulin sensitivity, reduced inflammation, and enhanced weight management among individuals adhering to plant-based diets [93,95]. The fiber content in these diets promotes satiety and aids in regulating blood glucose levels [94]. However, challenges related to nutrient adequacy, particularly vitamin B12, require consideration, and careful meal planning, are essential to consider [96].

High-protein, low-fat diets have garnered interest because of their potential to support satiety, weight loss, and glycemic control [96,97,98,99,100]. Clinical trials reveal that these diets may result in body weight decline, improved insulin sensitivity, and favorable changes in lipid profiles [96]. The emphasis on lean protein sources aligns with diabesity management goals, as protein helps preserve lean body mass [99]. However, long-term adherence to these diets can be challenging, necessitating ongoing support and monitoring [101].

Intermittent fasting regimens, including time-restricted feeding and periodic fasting, have gained popularity as potential strategies to enhance insulin sensitivity and promote weight loss [102,103,104]. Clinical trials suggest that these approaches can improve metabolic flexibility and support diabesity management [102]. Time-restricted feeding, in particular, restricts eating to specific time windows, potentially aligning with circadian rhythms and optimizing metabolic processes [103]. However, concerns regarding the sustainability of intermittent fasting regimens and the potential for disordered eating patterns necessitate further investigation [104].

One overarching theme emerging from the analysis of clinical trials is the importance of individualization in diabesity management [105,106,107]. The heterogeneity among individuals with diabesity underscores the need for personalized dietary interventions. Healthcare providers must consider individual preferences, metabolic profiles, and comorbidities when recommending specific diet types. A multidisciplinary approach involving healthcare providers, registered dietitians, psychologists, and patients is essential to address the multifaceted nature of diabesity [106]. Behavioral support, education, and ongoing monitoring play integral roles in achieving successful dietary interventions [107].

While clinical trials have provided valuable insights into diet types and diabesity management, several avenues for future research warrant exploration. First, elucidating the underlying mechanisms by which different diet types exert their effects on metabolic health is essential [Hall, Tay]. This knowledge can inform the development of targeted dietary interventions and personalized treatment plans. Second, exploring potential synergies between dietary interventions and pharmaceutical therapies may yield innovative approaches to diabesity management [108,109,110]. Finally, addressing the challenges of long-term dietary adherence remains a critical area for investigation [91,96,111].

**Figure 2:**
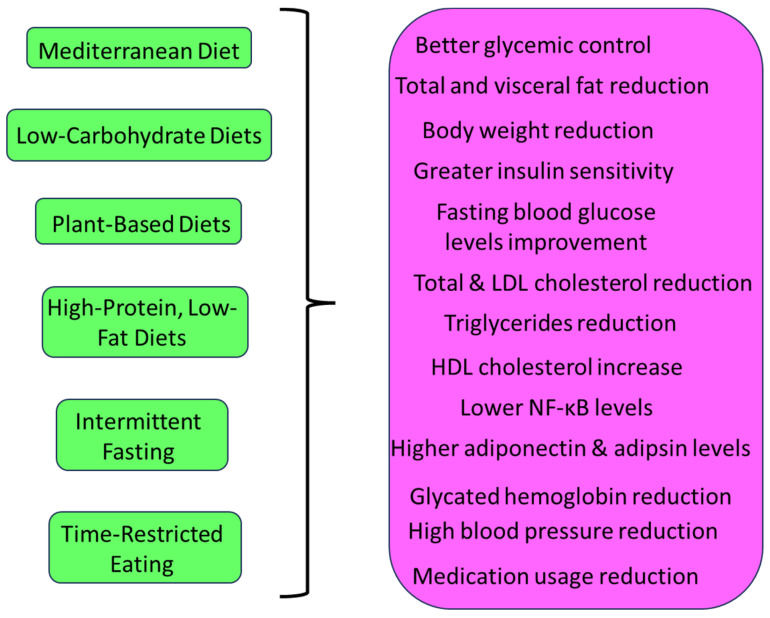
Diverse diets and their beneficial effects for diabesity management.

Collectively, the clinical trials reviewed in this article highlight the diverse landscape of diet strategies for diabesity management (Figure 2). While each diet type offers distinct advantages, individualization, long-term adherence, and holistic patient care are critical for effectively addressing the complex challenges posed by diabesity. The findings underscore the need for ongoing research, multidisciplinary collaboration, and patient-centered approaches to optimize diabesity management.

## 5. Conclusions

The present comprehensive review of clinical studies conducted over the past decade reveals a wealth of information on the impact of various diet types on diabesity, emphasizing the need for tailored dietary interventions. The MD constitutes the most well-studied diet, presenting also the most advantageous effects against diabetes and obesity, whereas no adverse side effects have been reported so far for this dietary pattern. Low-carbohydrate diets, particularly ketogenic diets, have demonstrated promising effects by improving glycemic control, reducing insulin resistance, and promoting weight loss among individuals with diabesity. However, long-term adherence and potential side effects require careful consideration. Plant-based diets, rich in fruits, vegetables, and whole grains as well as legumes, have demonstrated benefits in mitigating diabesity-related risks. They offer an array of nutrients and antioxidants that support metabolic health and weight management. High-protein, low-fat diets appear efficient in promoting satiety, weight decline, and glycemic control. These diets emphasize lean protein sources and limited fat intake, aligning with diabesity management goals. Intermittent fasting programs, such as time-restricted feeding and periodic fasting, have exhibited favorable outcomes in enhancing insulin sensitivity and metabolic flexibility. However, their suitability for long-term use necessitates further investigation.

The heterogeneity in diabesity necessitates a personalized approach to dietary interventions. Healthcare professionals should consider individual preferences, metabolic profiles, and comorbidities when recommending specific diet types. While various diet types can yield short-term benefits, sustained improvements in diabesity management require long-term adherence and lifestyle modifications. Behavioral support and education are essential components of successful interventions. Forthcoming studies must be focused on elucidating the mechanisms underlying the effects of different diet types on diabesity, exploring the potential synergies between dietary interventions and pharmaceutical therapies, and addressing the challenges of long-term dietary adherence. Managing diabesity effectively demands a multidisciplinary approach, involving healthcare providers, registered dietitians, psychologists, and patients. Collaboration and ongoing monitoring are integral to achieving optimal outcomes. Finally, patient-centered care remains paramount in diabesity management. Empowering individuals with the knowledge and skills to make informed dietary choices, in alignment with their unique needs and goals, is central to achieving lasting improvements in diabesity outcomes. Overall, this literature review underscores the multifaceted nature of diabesity and the potential of diverse diet strategies in its management. While each diet type offers distinct advantages, individualization, long-term adherence, and holistic patient care are critical for effectively addressing the complex challenges posed by diabesity.

## Data Availability

The data of the study are available upon request from the corresponding author. The data are not publicly available due to their potential future utilization by our research team.
